# Supplementary material for: Conditional chemoconnectomics (cCCTomics) as a strategy for efficient and conditional targeting of chemical transmission
Source: eLife. 2024 Apr 30;12:RP91927. doi: 10.7554/eLife.91927 (PMC11060718; doi:10.7554/eLife.91927)
Supplement: Figure 3—source data 3. [file elife-91927-fig3-data3.zip › Figure 3 source data3 part 1.zip!/Figure 3-Source data 3-part1/1175-9-CRISPResso_Report_rtnd62ko.zip!/CRISPResso_on_1175_12/2a.Nucleotide_percentage_quilt.pdf]

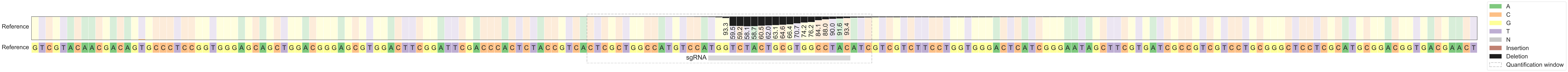

Reference
93.3
59.5
59.2
58.1
58.7
60.5
62.0
63.1
64.6
66.4
70.7
74.2
76.2
84.1
88.0
90.0
91.6
93.4
Reference
GTCGTACAACGACAGTGCCCTCCGGTGGGAGCAGCTGGACGGGAGCGTGGACTTCGGATTCGACCCACTCTACCGTCACTCGCTGGCCATGTCCATGGTCTACTGCGTGGCCTACATCGTCGTCTTCCTGGTGGGACTCATCGGGAATAGCTTCGTGATCGCCGTCGTCCTGCGGGCTCCTCGCATGCGGACGGTGACGAACT
sgRNA
A
C
G
T
N
Insertion
Deletion
Quantification window
